# The Intensity of Intensive Care: A Patient’s Narrative

**DOI:** 10.5539/gjhs.v4n5p20

**Published:** 2012-07-03

**Authors:** Alida Herbst, Cornelia Drenth

**Affiliations:** 1 School of Psychosocial Behavioural Sciences, North-West University, Potchefstroom Campus, South Africa; 2 Hospice Palliative Care Association – North West Hospices, South Africa

**Keywords:** intensive care unit (ICU), pre-eclampsia, illness narrative, psychosocial and spiritual implications, post traumatic stress

## Abstract

This qualitative study involved action research to explore one woman’s narrative of awareness, emotions and thoughts during treatment in an intensive care unit (ICU). The overarching aim is to increase insight into the thoughts, feelings and bio-psychosocial needs of the patient receiving treatment in ICU. Data was collected by means of narrative discourse analysis. Literature on the psychosocial and spiritual implications of ICU treatment is limited, and often patients have no recall of their treatment in an ICU at all. Documenting the illness narrative of this individual case is valuable as the participant could recall a certain amount of awareness, thoughts and emotions. These experiences included delirium, anxiety, helplessness, frustration and uncertainty. Once sedation was decreased, the patient’s consciousness increased and she was confronted with thoughts and emotions that were unrealistic and frightening. It was found in this study that the opportunity to share a narrative on the emotions and awareness during treatment in an ICU had cathartic value and the participant suffered little symptoms of post traumatic stress syndrome, often associated with long term treatment in an ICU. Further research on this topic is necessary to improve ICU treatment, not only on a physical level, but with emphasis on the psychosocial and spiritual needs of the patient.

## 1. Introduction

Many patients survive critical illness as result of rapidly evolving medical technology. Patients are usually only admitted to an intensive care unit (ICU) if they have a life-threatening illness. Medical treatment in an ICU always means exposure to a critical medical incident with associated physical and emotional trauma (Barclay, 2004). Most of the time, sedated patients are unaware of their environment and experience amnesia of the period in ICU. Some patients may experience pharmacology related withdrawal symptoms, anxiety, a feeling of suffocation, desperation to communicate and a lack of possibilities to flee (Jones, Humphris, & Griffiths, 1998 & 2000). Awareness and experiences while sedated are usually vague, but can often lead to post traumatic stress disorder (Barclay, 2004). Koshy, Wilkinson, Harmsworth and Waldmann (1997) found that up to 15% of patients with a stay over 4 days in ICU were suffering from post-traumatic stress disorder (PTSD) a year after discharge. Skirrow, Jones, Griffiths and Kaney (2001) are of the opinion that a significant proportion of ICU patients experience significant psychological distress for months and sometimes years after hospitalization.

Illness and trauma are challenges that involve the individual as a totality. Little is known about the psychosocial dynamics of a patient who recovers from critical illness and the psychological impact of the ICU experiences have not been fully investigated (Johnston, 2011). It is only since the 1990’s that authors began to describe the delirium, hallucinations and post-traumatic stress disorder symptoms experienced by ICU patients (Johnston, 2011). Dealing with the complex bio-psychosocial and spiritual processes involved in recovering from severe illness and ICU treatment requires multi-disciplinary intervention. This research has the potential to increase sensitivity among behavioral scientists, health care and spiritual workers for the unique contextual experiences of the patient treated in an ICU.

Every individual’s specific context determines how illness and trauma will be experienced, leading to consideration of an ethical epistemology to be considered in research (Kelley, 2002; Truter, 2002). Insight in the narrative context of illness or *illness scripts* (Biswas, Sarkar, Theodore, Parajuli, Alurkar, Shetty & Nagra, 2002) has the potential to supply a framework for holistic patient care by enriching psychosocial and spiritual knowledge and research. Sometimes the illness stories of patients are ignored, because “these voices are often faltering in tone and mixed in message” and “these voices bespeak conditions of embodiment that most of us would rather forget our vulnerability to” (Frank, 1995:25). People’s vulnerability towards illness and their own mortality often affect their perceptions of themselves, their spirituality and their experience of God. The spiritual worker in the health care setting respects the individual’s experience of illness. By following a contextual narrative approach, the individual is guided to reconstruct trauma into a life enriching experience (Truter, 2002). The telling of life stories can play a key role in the healing of trauma (Haarhoff, 1998). Kelley (2002) and White and Epston (1990) confirm that the narrative approach can help individuals to find meaning in life experiences. According to Stuhlmiller (2001:66) *healing* and *meaning* can be seen as *therapeutic transformation*, something that will lead to change. On the one hand the research participant is considered as an expert who had an experience that scientists can learn from, and on the other hand the individual is guided to find meaning in illness and to grow from the experience. Davis (2001) refers to this as “post-traumatic growth”.

### 1.1 Goal and Objectives

The overarching aim of this study is to explore the narrative of awareness, emotions, thoughts and spiritual needs of an individual who received treatment in an ICU in an attempt to reconstruct the realities, help the individual find meaning in the experience and to obtain information on the psychosocial and spiritual needs of patients in an ICU. This aim resulted in the following objectives:

- To allow the research participant reflection on the period in ICU through sensory awareness and externalization
- To reconnect experiences with reality
- To internalize the experience
- To find meaning in the experience
- To make recommendations regarding the psychosocial and spiritual needs of the ICU patient

## 2. Method

A qualitative research design (Fouché & Delport, 2005) was followed and data rich in description was collected from a one shot case study (Fouché, 2005). This method involves purposive sampling of a single research unit to be observed and interviewed for a specific period and the collection of all relevant data. The background history is important as it is a very individualized type of research and results cannot be generalized. Denzin and Lincoln (1994) and Henning, Van Rensburg and Smit (2004) emphasize that qualitative research is not a rigid investigation and measurement of quantity, intensity or frequency, but a focus on realities as defined by the research unit. The aim is not to interpret or to prove, but to supply a safe environment for individuals to express their experiences. Toombs (1992) refers to this process of expressing one’s illness story as *illness-as-lived*. The information collected is not necessarily objective. Frank (1995) reacts on the objectivity of data by differentiating between *disease* and *illness*:

*Objective talk about disease is always medical talk. When a person becomes a patient and learns to talk disease talk, her body is spoken as a place that is elsewhere, a ‘site’ where the disease is happening. If disease talk measures the body, illness talk tells of fear and frustration of being inside a body that is breaking down. In illness-talk there is no such thing as the body, only my body as I experience it. What is happening to me? Not it, but me*.

Taking this opinion into consideration, the researcher’s role is not to interpret or to prove, but to supply an environment for illness talk. Stringer (1999:208) describes the researcher as a “scribe-for-the-other to give voice to their own interpretations of events, working with them to identify the key elements of their experience and shape them into a report”. Both the researcher and the research participant are involved in a process of giving meaning. All conversations were recorded to assist the researcher in data analysis. Due to limited space, only excerpts from the dialogue will be included in this article.

Narrative conversations with the research participant started immediately after discharge from the ICU until six months after her discharge from hospital. De la Porte’s (2004) guidelines for narrative conversations after trauma were integrated with Britten’s (2000) suggestions for questions in qualitative research to guide the in-depth interviews. The following four phases guided the process:

- Externalization of the trauma (The landscape of action)
- Internalization of survival (The landscape of identity)
- Doing hope
- Closure

It is important to emphasize that narrative research it is not only the exchange of stories and re-writing it in a creative journaling style, but to scientifically and systematically identify themes that could be analyzed and evaluated (Overcash, 2003). Data was analyzed and interpreted through the steps of selective coding in the grounded theory described by De Vos (2005).

### 2.1 Narratives of Illness

A narrative can be defined as a story or “…a number of events, linked in sequence, across time, according to a plot” (Morgan, 2000). Narrative work aims to help individuals to understand their experiences better, to identify the unique outcomes of their stories and to change the effect of trauma on their lives by focusing on a growth directed outcome (De la Porte, 2004). According to Kelley (2002) narratives assist the client in deconstructing the story lines, assessing the plot, characters and time line for meaning, and then moves on to search for other existing truths. It may seem that narrative work is a very simple process of story-telling. In this regard, Riessman (1994:114) emphasizes that “Stories are a kind of cultural envelope into which we pour our experiences and signify its importance to others, and the world of the story requires protagonists, inciting conditions and culminating events”.

The multidisciplinary field of narrative medicine offers paths to better understanding critical illness experience and tools to help the patient (Stanley & Hurst, 2011). Narratives of illness can help with the physical, mental and spiritual healing of the patient and can play a key role in the healing of trauma (Haarhoff, 1998). According to Biswas et al. (2002), illness scripts have the following advantages:

- The process of getting ill, being ill, getting better or worse, and coping or failing to cope, can be thought of as enacted narratives within the wider narratives of people’s lives
- Narratives of illness provide a framework for approaching a patient’s problem holistically and other diagnostic and therapeutic options may be uncovered
- Taking a history and the interpretation of it is central to the analysis of narratives
- Narratives offer a method for addressing existential qualities such as inner hurt, despair, hope, grief, moral pain and may even constitute people’s illness
- The lost tradition of narratives in the practice of medicine can be revived

### 2.2 Background Information of the Participant

The 27 year old Francine Nothnagel^1^ was in the last trimester of her pregnancy when she was admitted to hospital due to dangerously high blood pressure. She did not feel sick and was surprised when the doctor diagnosed pre-eclampsia, a condition that occurs after the 20^th^ week of pregnancy causing high blood pressure and increased protein in the mother’s urine. This condition affects the placenta, the mother’s kidneys, liver and brain with increased risk of mortality for the mother and complications to the fetus (Pre-eclampsia Foundation, 2005). Francine’s husband, Christo, who was working in Iraq at that time, had to return to South Africa urgently to be with his wife. She was taken to the operating theatre for an emergency caesarian section. Francine’s last memory of that day was that the spinal injection had no effect, that she still had feeling in her legs and that the doctor told her he had to give her a general anesthetic.

In the operating theatre, it was literally a matter of life and death. Francine suffered from severe bleeding and she had to receive several blood transfusions. The doctor had to do a total hysterectomy. The radical surgery and multiple blood transfusions lead to further complications: adult respiratory distress syndrome (ARDS) and kidney failure (Mostert, 2004). She was transferred to the ICU in a critical condition. Mortality was estimated at 30-50% (Mostert, 2004) and her family was prepared that she could possibly die.

Francine’s baby, Neil, was born prematurely and was in a stable condition in the neo-natal ICU. Her husband and her mother were with the two patients most of the time. During her treatment in ICU, Francine was ventilated and sedated, but she experienced severe anxiety, hallucinations and awareness that were beyond her comprehension. She made a miraculous recovery and against all predictions, she survived. The sedation was gradually stopped and after three weeks in the ICU, she regained consciousness. She was disorientated and anxious and wanted to find meaning in her thoughts, feelings, dreams and other awareness. She constantly verbalized a feeling that her whole stay in the ICU was unreal and vague. She learned from her family and the nursing staff how critical her condition actually was, but she felt excluded from the whole ordeal. She was determined to tell her story in an attempt to give meaning to her experiences.

## 3. Results

The results will be presented as a summary of the different phases of the narrative process that was followed with the participant.

### 3.1 Reflections on Experiences in the ICU (Externalization)

This conversation took place shortly after Francine was transferred from the ICU to the general ward. She was fully conscious, very emotional and traumatized by the whole situation. Francine was allowed to share her story with the researcher as facilitator and active listener and without interruption. The aim was to lead her to self-exploration of “…inner experience from the private sphere into the semi-public domain of validation and worth” (Stuhlmiller, 2001: 67).

Francine could remember being taken to the operating theatre and that the spinal injection did not work. Her last active memory was the doctor’s words that he had to give her a general anesthetic. The next thing that she could remember was about two to three weeks later when she heard from everyone that she was practically dead and that her survival was actually a miracle.

Her thoughts were vague and chaotic. She remembered having the sense of a family that was taking care of her, but they were strangers. They had very naughty children and the man of the house was in the bath most of the time. She had a constant need to empty her bladder, but could not enter the toilet due to the man being in the bath all the time. The children used to smoke next to her and that made her very anxious and angry because she was pregnant. The whole family ignored her and continuously sprayed some deodorant that was in a white polystyrene pipe.

She regularly dreamt of Christo and wanted to go home to take a shower. She even tried to bribe the doctor in an attempt to get home. At one stage she had an image of herself being the sun, rising on the horizon. The next moment a huge lawnmower appeared and started to cut her hair. She often referred to a feeling of being frozen and seeing moths all over her room. She had images of being on an airplane of Air France, being connected to some sort of pipe all the time and having a baby in her shirt’s pocket. Inside the airplane was lots of green grass and her sister-in-law was there in a bunny-suit. The airplane could not land and passengers jumped from plane to plane with parachutes. Christo used a missile and a parachute to get off the plane.

Various other memories were recounted, most of them irrational, vague and distorted. Some of the prominent memories were about a small, bloody, purple thing in a plastic bag and huge, poisonous frogs in her room. The frogs then became crocodiles that constantly made a suctioning and blowing sound. There was spaghetti hanging from the ceiling that would rot if it is too warm. She could not remember when she started to realize that she was in hospital, but she was aware that something must have happened to her and the baby. She had memory of the following words: “Don’t worry, the baby is alive”. Halucinatory experiences appear to be common for ICU patients. Johnston (2011:373) describes these experiences as “a developmental mechanism which the patient’s mind is using to process psychological issues”. Francine’s first memories after discharge from the ICU corresponds with literature (Johnston, 2011; Jones, Griffiths, & Humphris, 2000; Papathanassoglou & Patikari, 2003; Richman, 2000; Wallen, Chaboyer, & Thalib, 2008).

### 3.2 Central Themes Identified from the First Conversation

The central themes that were identified from the first conversation are summarized in Table 1. The main themes were chaos and delusion, helplessness and inability, mortality and injury, social support, hopeful messages, loneliness and isolation. We decided to focus on Francine’s dreams, delusions and surrealistic experiences as the main topics of our next conversation because Francine experienced them as frightening and vague recollections that increased her anxiety and insecurity.

**Table 1:** Central themes from the first conversation

Themes from the Conversation	Actual Words or Phrases Used by The Participant	Possible Links with the Situation
- Helplessness and inability	- *Couldn’t* - *Unable* - *Not allowed* - *In a wheelchair* - *In bed*	Paralysis and total care dependency while being sedated and treated in an ICU

- Mortality and injury	- *Dead / died* - *Disappeared* - *The other child was in hospital* - *A small, bloody purple thing in a plastic bag* - *Myself lying in a refuse bin* - *I saw blood* - *Poisonous frogs*	Estimated mortality of the study unit was between 30 and 50% due to severe blood loss and adult respiratory stress syndrome

- Loneliness and isolation	- *They were not my family* - *They ignored me* - *My mother-in-law looked like someone else* - *Christo was with another boy* - *The boy with the blonde hair disappeared*	Isolation associated with ICU treatment and being sedated

- Specific emotions	- *Anxious* - *Angry*	Frustration associated with being care dependant

- Chaos and delusion	- *Vague* - *Chaotic* - *The children used to smoke next to me* - *Deodorant that was in white polystyrene* - *Wanted to cut the grass* - *I was on an airplane of Air France* - *My baby, Neil, was in my shirt’s pocket* - *Moths all over my room* - *My sister-in-law … in a bunny-suit of all things!* - *I had twins, but the one died* - *A farm between Brits and George* [these two towns are almost 1500km from each other] - *Passengers jumped from plane to plane with parachutes* - *Christo used a missile and a parachute to get of the plane* - *Paid only 1 c for the house!* - *There was spaghetti on the ceiling* - *I was on the Linden School Bus* - *Christo was back on the plane in an Iraq Uniform* - *Crocodiles that constantly made a suctioning and blowing sound*	Delusion and hallucinations are often associated with sedatives used in ICU treatment Francine was pregnant at the last stage of her conscious awareness Her husband was in Iraq for contract work and had to urgently fly to South Africa to be with his wife

- Social support systems	- *Christo* - *My baby, Neil, pregnant and caesarian section* - *My mother-in-law* - *My sister-in-law* - *My father*	Francine’s husband and other family members were with her as often as possible

- Hopeful messages	- *Miracle* - *Survived* - *A family taking care of me* - *Lots of green grass* - *The house of my dreams* - *I was delighted* - *Don’t worry, the baby is alive!*	Family and medical staff constantly talked next to her bed and with her about her recovery and the baby

### 3.3 Exploring Sensory Awareness and Connecting Experiences to Reality

Francine was still recovering, but had an urge to go back to the ICU in an attempt to understand what had happened to her. The nursing staff reconstructed the ICU room where Francine was treated to look almost the same as it did when she was there. This is a process of connection and is described by Stuhlmiller (2001: 67) as an “…opportunity to work out puzzles and concerns…”. The researcher had the important role of helping the narrator find meaning through the clarification of experiences. Francine’s mother and the nursing staff were involved in the conversation and the process of connecting experiences to reality. Francine was allowed to first just look through the ICU environment and was encouraged to share any awareness or experiences that she could recall at that specific time. She started to tell more about her story. She managed to identify certain sounds, smells and images and asked various questions. The image of a baby in her shirt’s pocket was clarified with the nurse’s answer that they brought baby Neil to her on a daily basis and placed him on her chest to stimulate his development and improve bonding. The sound of the airplane was similar to the sound of the air conditioner in the room. She also pointed out that the ventilator made the suctioning and blowing sounds that she remembered the crocodiles in her dreams made. Francine also remembered her mother asking why her room was so cold and that the nursing staff answered it was necessary to keep the room at about 18° C to reduce the risk of infection. Francine linked these conversations on the temperature to the spaghetti on the ceiling. All the lines (plastic tubes for intravenous medicine and feeding) that hung above her bed could have looked like spaghetti. In her distorted mind, she interpreted the risk of infection as the spaghetti that would rot in the heat. The white polystyrene pipe and the smoking turned out to be the daily physiotherapy sessions where she had inhalations to loosen the phlegm from her lungs. The constant need to empty her bladder was clarified when the nurse explained that a urinary catheter often causes the sensation of a full bladder.

### 3.4 Looking Back: Reflections on Beliefs, Knowledge and Feelings of Survival (Internalization)

After we worked through the background and Francine’s memories and uncertainties, the theme of our next conversation was survival. In this section, the actual dialogue is quoted to increase authenticity. FN refers to Francine, while AH refers to one of the researchers.

AH: Francine, now that you have a better background of what has happened to you and how ill you really were, how do you think you survived through all of this?

FN: I really don’t know. My thoughts were so much confused, but I think it was my faith and the support of my family that pulled me through. Everyone tells me I made a miraculous recovery.

AH: Do you think you had anything to do with your survival?

FN: I’m not sure. Maybe it is because of my age and that I was in good health.

AH: On a more spiritual-emotional level – do you think you had anything to do with your survival?

FN: I think that I desperately fought back in my thoughts. I argued with the people who smoked. I was concerned that the spaghetti would rot and I would die. I wanted to keep the baby in my pocket. I am very strong willed!

AH: How did your thoughts and strong will help you?

FN: I constantly thought about Neil and Christo. I was aware of my mother talking and praying and I knew she wouldn’t let me die without a fight. I had to fight and I was wondering if Neil was okay.

AH: Can you remember any questions in your mind that you tried to resolve?

FN: I wanted to know Neil’s birth weight. And I wanted to be clean and have my hair cut.

AH: It seems as if you had certain thoughts that kept you going. Do you think that your emotions had anything to do with the way you lived through this crisis?

FN: I can’t remember any other emotions but anxiety. At the moment I am struggling to feel anything. It is as if it didn’t happen to me. It still feels unreal.

AH: Maybe you can tell me more about how *unreal* feels.

FN: I don’t know much about the drama around me, how critical my condition was or that I nearly died. I have to hear this from others – you, my mother, Christo, the nursing staff. I think under normal circumstances, I am a fighter, but I feel excluded from what happened in ICU.

AH: Although you may feel excluded, we managed to identify at least three things inside yourself that helped you survive: your faith, strong will and fighting spirit. Are you proud that you survived?

FN: Actually, all I feel is gratitude – because Neil is healthy and I survived. God must have further plans with our lives! It is just that the hysterectomy is another unreal feeling. I can’t believe that I won’t be able to have more babies, but that makes Neil even more precious.

AH: You suffered trauma and loss, but you express gratitude, you seem to be quite calm and you are verbalizing your thoughts and feelings freely. How do you think your life prepared you to survive a crisis like this one?

FN: My faith, my mother and my husband helped me through this.

AH: But do you think that your past and life in general had any influence on the way you survived?

FN: I have a sister who is cerebral palsied. She is care dependant and I help my mother to take care of her. It taught me about life, how precious it is and that I have to take responsibility for my life and the people in my life. It created a very special bond between my mother and me.

AH: How did your recent trauma contribute to your collection of life lessons?

FN: I have a deeper perspective on life. I realize that life is not in our hands - that God is in control. I realize how precious one’s family is. Christo, Neil and my parents are the most important people in my life. I don’t want Christo to return to Iraq. His presence here is more important than money and financial freedom.

### 3.5 Finding Meaning (Doing Hope)

Through internalization of survival, it was possible to turn Francine’s trauma into an existential triumph of survival, leading the narrative process into the phase of doing hope. Excerpts from the actual dialogue are included again to recognize the research participant’s own interpretation of hope.

AH: Francine, we decided to focus on hope during today’s conversation. How do you plan to keep hope alive after what you have learned from your recent trauma?

FN: I gave a lot of thought to the concept of hope. I believe it is something that one can only get from God – He is my hope.

AH: Your faith played a very important role in your survival and in establishing hope. Do you think that faith is the only aspect that helped you to maintain hope through this trauma?

FN: No. I can remember Christo telling me: *Open your eyes!* To open my eyes, to see Christo again, to see my baby, that was my hope.

AH: Can you think of previous experiences in life that influenced the way you look at hope?

FN: I think I have learned all about hope from my disabled sister and the way my mother treats her as *normal*. I will never forget how she takes care of her and how much faith my mother has in the Lord. That is where I learned that hope and God are one.

AH: Who or what keeps hope alive?

FN: Christo, my mother and God keep my hope alive. Now I also have Neil – he is my miracle, the ultimate hope.

AH: How do you think your hope will help you in the future?

FN: I have lost my uterus and it somehow stole a bit of my hope of having another child, but I have Neil. He is my miracle, my love child. I am sincerely thankful to the Lord who promised that He will bless us and keep us. I have Christo. We are a family.

AH: The loss of your uterus stole a bit of your hope. Can you think of any other *hope thieves* in your life?

FN: The hysterectomy is still a bit of a shock. I am scared that I will start bleeding again or that something will happen to Neil. It is mostly a feeling of fear, not anxiety or hopelessness. I will be very careful, but I want to go on with my life. I want to be with my family and enjoy every moment of it.

### 3.6 Personal Growth: Turning Circumstances into Opportunities

The aim of this conversation was to let Francine reflect on the spiritual and emotional growth that resulted from her trauma. Although she was struggling to deal with the impact of the hysterectomy, she was in a process of searching for meaning. She described the search for meaning as a life long process where every new experience has new challenges resulting in redefining meaning. The most important life lessons that she had learned was that life is unpredictable, than everyone is mortal, that dying isn’t that easy and that nobody knows about dying – only God. She also learned that she had the ability to survive serious trauma through the grace of God. She was intensely aware of the fact that God is in control of her life and that the love and support of her husband and mother were the other forces that positively influenced her survival. She also felt that this experience empowered her to face future challenges and realities. This experience of growth is described by Papathanassoglou and Patiraki (2003:20) in the following words: “Critical illness may entail psychological (inner consciousness) processes and it may stimulate individual growth, instead of being merely a profoundly unpleasant state through which we need to navigate the individual…”

The main theme of this conversation was gratitude. Francine described gratitude in the following words: “Gratitude is a very special feeling – I have learned that nothing in life can be taken for granted. It is precious to be healthy, to be alive, to have a family, to have God”. On a question on how Francine intended to celebrate her survival, she answered that Neil’s birthday will be her personal festival of gratitude every year. The following is an excerpt from our final conversation:

AH: Do you think that the whole ordeal will influence your future?

FN: Definitely. I won’t live my dream of having more children, but on a spiritual level I have grown to new insights and maturity. I look at life differently. Christo is coming back from Iraq soon. I am not going back to a full-time job, because I want to spend enough time with Neil. I will have only this one chance to live with him through his baby and toddler years. I don’t want to miss out. I realize now what the true value of life is. My life is much more valuable than it was two years ago.

### 3.7 Closure

Francine was followed up in two more individual sessions until about six months post-trauma. There were no signs of posttraumatic stress syndrome and she and her family managed to maintain a healthy and balanced psychosocial and spiritual functioning.

## 4. Recommendations

Following Francine’s experiences and awareness during treatment in an ICU, some recommendations can be made to increase the multi-disciplinary team’s sensitivity towards sedated or unconscious patients. From a bio-medical viewpoint, these recommendations may not be scientifically meaningful, but for behavioral scientists or spiritual workers in the health care environment, these recommendations hold the potential to increase quality of life and to reconstruct trauma in a life enriching experience.

- Consider the possibility that the patient can hear all conversations in the room, although absorption of information may be distorted.
- The patient needs reassurance, compassion and care.
- Explain medical and other procedures to the patient.
- Keep the patient informed about everyday realities like the time of day and what the weather is like outside.
- Touch the patient in a comforting way.
- Talk to the patient continuously in a comforting and reassuring way.
- Provide spiritual support to the patient and constantly communicate hopeful messages to the patient.
- Ask the family to take photos of the patient while in ICU – it can help the person to feel more “included” in their own trauma and experiences once they have recovered.
- Inspiring music in the background may reduce common sounds in the ICU (e g the ventilator). Consult with the family to determine the patient’s musical preferences.

## 5. Limitations and Contribution of the Study

This study reports on a single research unit’s experiences and the results can in no way be generalized to the broader population of patients receiving treatment in an ICU. The narrative process is subjective in the sense that it reflects the personal views, perceptions, interpretations and experiences of the individual, and although these were deconstructed and reconstructed in such a way that it was closer to the realities faced in the situation described through this study, not everything can be applied to other patients in similar situations. The greatest contribution of this study is that it could assist health care professionals in their understanding of the psychosocial needs and awareness of patients in an ICU.

## 6. Summary and Conclusion

Although this was a one-shot case study and findings cannot be generalized, it is clear from this study that patients being treated in an ICU can be aware of various experiences even when they are heavily sedated. As drawn from the literature, patients in ICU may struggle with delusions, helplessness, mortality, loneliness, isolation and anxiety, but may also experience hope, faith, inner strength and social support. In this case the research unit’s inner strength, faith and social support greatly contributed towards her recovery. Through narrative conversations, the patient can be assisted to construct, deconstruct and reconstruct his/her experiences and find hope and meaning. Telling one’s narrative of experiences in an ICU has the potential to minimize the risk of developing post traumatic stress disorder and can lead to social, emotional and spiritual growth.

## Footnotes

1 The research participant’s name is used in this article with her informed and written consent and her contribution is hereby acknowledged.
